# Complete genome sequence of *Thalassolituus oleivorans* R6-15, an obligate hydrocarbonoclastic marine bacterium from the Arctic Ocean

**DOI:** 10.4056/sigs.5229330

**Published:** 2014-03-01

**Authors:** Chunming Dong, Xin Chen, Yanrong Xie, Qiliang Lai, Zongze Shao

**Affiliations:** 1 Key Laboratory of Marine Genetic Resources, Third Institute of Oceanography, State Oceanic Administration, Xiamen, China;; 2 State Key Laboratory Breeding Base of Marine Genetic Resources, Xiamen, China;; 3 Key Laboratory of Marine Genetic Resources of Fujian Province, Xiamen, China; .; 4 Life Science College, Xiamen University, Xiamen 361005, Fujian, China.

**Keywords:** *Thalassolituus*, genome, alkane-degrading, surface seawater, Arctic Ocean

## Abstract

Strain R6-15 belongs to the genus *Thalassolituus*, in the family *Oceanospirillaceae* of *Gammaproteobacteria*. Representatives of this genus are known to be the obligate hydrocarbonoclastic marine bacteria. *Thalassolituus oleivorans* R6-15 is of special interest due to its dominance in the crude oil-degrading consortia enriched from the surface seawater of the Arctic Ocean. Here we describe the complete genome sequence and annotation of this strain, together with its phenotypic characteristics. The genome with size of 3,764,053 bp comprises one chromosome without any plasmids, and contains 3,372 protein-coding and 61 RNA genes, including 12 rRNA genes.

## Introduction

*Thalassolituus* spp. belong to the *Oceanospirillaceae* of *Gammaproteobacteria*. The genus was first described by Yakimov *et.al.* (2004), and is currently composed of two type species, *T. oleivorans* and *T. marinus* [1,2]. Bacteria of this genus are known as obligate hydrocarbonoclastic marine bacteria [3]. Previous reports showed that *Thalassolituus*-related species were among the most dominant members of the petroleum hydrocarbon-enriched consortia at low temperature [4-7]. In addition to consortia enriched with oil, *Thalassolituus* spp. can be detected in variety of cold environments as well [8-10].

Strain R6-15 was isolated from the surface seawater of the Arctic Ocean after enriched with crude oil during the fourth Chinese National Arctic Research Expedition of the “*Xulong*” icebreaker in the summer of 2010. The 16S rRNA gene sequence shared 99.86% and 96.39% similarities with *T. oleivorans* MIL-1^T^ and *T. marinus* IMCC1826^T^, respectively. Pyrosequencing results (16S rRNA gene V3 region) of fifteen oil-degrading consortia across the Arctic Ocean showed that the dominant member in most of the consortia shared identical sequence of this strain, comprising 8.4-99.6% of the total reads (not published).

Here, we described the complete genome sequence and annotation of strain *T. oleivorans* R6-15, and its phenotypic characteristics. Moreover, a brief comparison was made between strain R6-15 and the two type strains of the validly named species of this genus, in both phenotypic and genomic aspects.

## Classification and features

**Figure 1:**
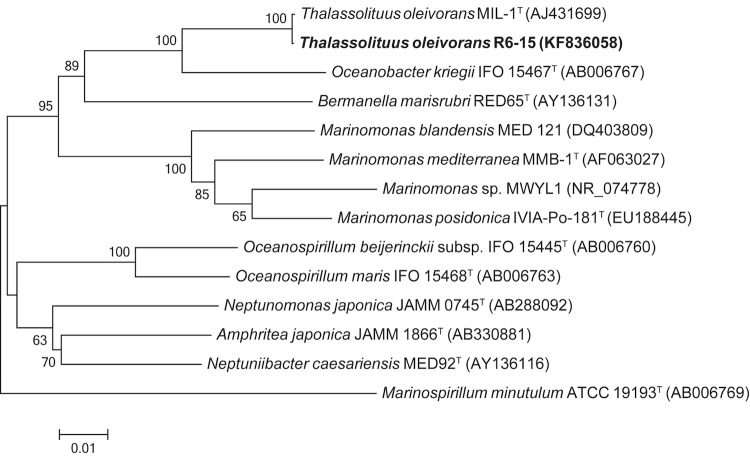
Phylogenetic tree highlighting the position of *T. oleivorans* strain R6-15 relative to other type and non-type strains with finished or non-contiguous finished genome sequences within the family *Oceanospirillaceae*. Accession numbers of 16S rRNA gene sequences are indicated in brackets. Sequences were aligned using DNAMAN version 6.0, and a neighbor-joining tree obtained using the maximum-likelihood method within the MEGA version 5.0 [11]. Numbers adjacent to the branches represent percentage bootstrap values based on 1,000 replicates.

**Figure 2:**
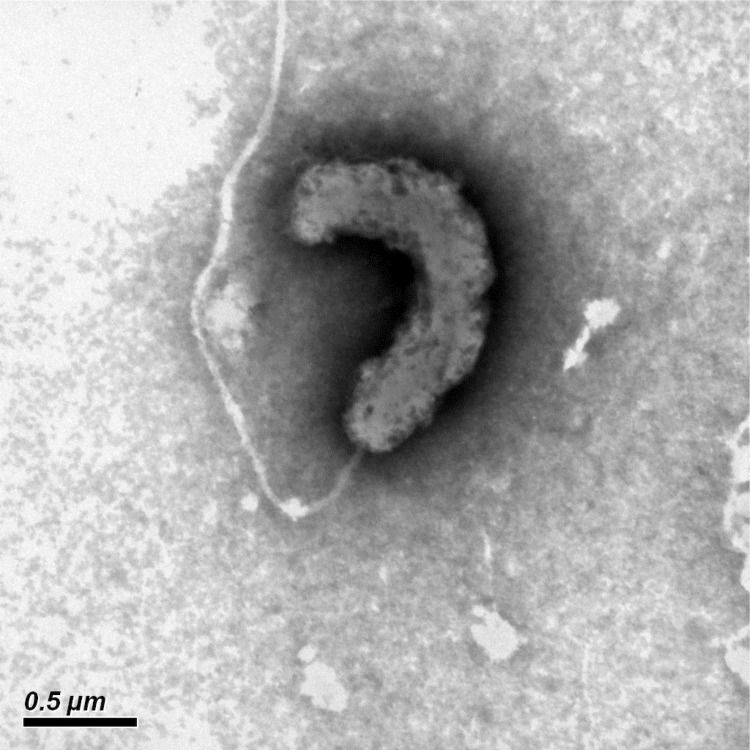
Transmission electron micrograph of *T. oleivorans* R6-15, using a JEM-1230 (JEOL) at an operating voltage of 120 kV. The scale bar represents 0.5 µm.

*T. oleivorans* R6-15 is closely related with *T. oleivorans* MIL-1^T^ (Figure 1, Table 1). The strain is aerobic, Gram-negative and motile by a single polar flagellum, exhibiting a characteristic morphology of a curved rod-shape cell (Figure 2). Strain R6-15 is able to utilize a restricted spectrum of carbon substrates for growth, including sodium acetate, Tween-40, Tween-80 and C12-C36 aliphatic hydrocarbons. Its growth temperature ranges from 4 to 32°C with optimum of 25°C.

**Table 1:** Classification and general features of *T. oleivorans* R6-15 according to the MIGS recommendations [12].

**MIGS ID**	**Property**	**Term**	**Evidence code**^a^
		Domain *Bacteria*	TAS [13]
		Phylum *Proteobacteria*	TAS [14]
		Class *Gammaproteobacteria*	TAS [15-17]
	Current classification	Order *Oceanospirillales*	TAS [16,18]
		Family *Oceanospirillaceae*	TAS [16,19]
		Genus *Thalassolituus*	TAS [1]
		Species *Thalassolituus oleivorans*	IDA
	Gram stain	Negative	IDA
	Cell shape	Curved rods	IDA
	Motility	Motile	IDA
	Sporulation	Non-sporulating	IDA
	Temperature range	4-32°C	IDA
	Optimum temperature	25°C	IDA
	Carbon source	Sodium acetate, Tween-40, Tween-80, alkanes (C12-C36)	IDA
	Energy source	Chemoorganotrophic	IDA
	Terminal electron receptor	Oxygen	IDA
MIGS-6	Habitat	Surface seawater	IDA
MIGS-6.3	Salinity	0.5-5% NaCl (w/v)	IDA
MIGS-22	Oxygen	Aerobic	IDA
MIGS-15	Biotic relationship	Free-living	IDA
MIGS-14	Pathogenicity	Unknown	NAS
MIGS-4	Geographic location	Chukchi Sea, Arctic Ocean	IDA
MIGS-5	Sample collection time	July 2010	IDA
MIGS-4.1	Latitude	69°30.00′	IDA
MIGS-4.2	Longitude	-168°59.00′	IDA
MIGS-4.3	Depth	Surface seawater	IDA
MIGS-4.4	Altitude	Sea level	IDA

a) Evidence codes - IDA: Inferred from Direct Assay; TAS: Traceable Author Statement (i.e., a direct report exists in the literature); NAS: Non-traceable Author Statement (i.e., not directly observed for the living, isolated sample, but based on a generally accepted property for the species, or anecdotal evidence). These evidence codes are from the Gene Ontology project [20]. If the evidence code is IDA, then the property should have been directly observed, for the purpose of this specific publication, for a live isolate by one of the authors, or an expert or reputable institution mentioned in the acknowledgements.

When compared to other *Thalassolituus* species, strain R6-15 differed from type strain MIL-1^T^ [1] in catalase, urease and acid phosphatase, and in the utilization of *n*-alkane, pyruvic acid methyl ester, D-mannitol and D-sorbitol (Table 2). Differences were also observed with type strain IMCC1826^T^ [2] in growth temperature range, catalase, nitrate reductase, urease and leucine arylamidase and the utilization of n-alkane, pyruvic acid methyl ester, β-Hydroxybutyric acid and D,L-Lactic acid (Table 2).

**Table 2:** Differential phenotypic characteristics between *T. oleivorans* R6-15 and other *Thalassolituus* species.

**Characteristic**	**1**	**2**	**3**
Cell diameter (µm)	0.25-0.4 x 1.2-2.0	0.32-0.77x1.2-3.1	0.4-0.5 x1.2-2.5
Salinity/Optimum (w/v)	0.5-5%/ 3%	0.5-5.7%/ 2.3%	0.5-5.0%/ 2.5%
Temperature range (°C)	4-32	4-30	15-42
Number of polar flagella	1	1-4	1
**Production of**			
Catalase	-	+	+
Nitrate reductase	-	-	+
Urease	w	-	+
Acid phosphatase	+	-	+
Leucine arylamidase	+	+	-
**Carbon source**			
Sodium acetate	+	+	na
n-alkane	C12-C36	C7-C20	C14 and C16
Pyruvic acid methyl ester	w	-	+
β-Hydroxybutyric acid	-	-	+
D,L-Lactic acid	-	-	+
D-Mannitol	-	+	-
D-Sorbitol	-	+	-
Geographic location	Chukchi Sea, Arctic Ocean	Harbor of Milazzo, Italy	Deokjeok island, Korea
Habitat	surface seawater	seawater/sediment	surface seawater
G+C content (mol%)	46.6	46.6	54.6

Strains: 1, *T. oleivorans* R6-15; 2, *T. oleivorans* MIL-1^T^; 3, *T. marinus* IMCC1826^T^. +: positive result, -: negative result, w: weak positive result, na: data not available.

## Genome sequencing information

### Genome project history

This organism was selected for sequencing on the basis of its phylogenetic position and dominance position in the crude oil-degrading consortia enriched from the surface seawater of the Arctic Ocean. The complete genome sequence was deposited in Genbank under accession number CP006829. Sequencing, finishing and annotation of the *T. oleivorans* R6-15 genome were performed by the Chinese National Human Genome Center (Shanghai). Table 3 presents the project information and its association with MIGS version 2.0 compliance [21].

**Table 3:** Project information

**MIGS ID**	**Property**	**Term**
MIGS-31	Finishing quality	Finished
MIGS-28	Libraries used	one 454 pyrosequence standard library
MIGS-29	Sequencing platforms	454 GS FLX Titanium
MIGS-31.2	Fold coverage	21.1 ×
MIGS-30	Assemblers	Newbler version 2.7
MIGS-32	Gene calling method	NCBI PGAP pipeline
	GenBank ID	CP006829
	GenBank Date of Release	On publication
	GOLD ID	Gi20060
	Project relevance	Crude oil-degradation, biogeography

### Growth conditions and DNA isolation

Strain R6-15 was grown aerobically in ONR7a medium [22] with sodium acetate as the sole carbon and energy source. The genomic DNA was extracted from the cell, concentrated and purified using the AxyPrep bacterial genomic DNA miniprep Kit (Axygen), as detailed in the manual for the instrument.

### Genome sequencing and assembly

The genome was sequenced by using a massively parallel pyrosequencing technology (454 GS FLX) [23]. A total of 140,550 reads counting up to 78,223,504 bases were obtained, covered 21.1-folds of genome. The Newbler V2.7 [24] software package was used for sequence assembly and quality assessment. After assembling, 64 contigs ranging from 500 bp to 304,980 bp were obtained, and the relationship of the contigs was determined by multiplex PCR [25]. Gaps were then filled in by sequencing the PCR products using ABI 3730xl capillary sequencers. A total of 284 additional reactions were necessary to close gaps and to raise the quality of the finished sequence. Finally, the sequences were assembled using Phred, Phrap and Consed software packages [26], and low quality regions of the genome were re-sequenced. The final sequence accuracy was approximately 99.999%.

### Genome annotation

The protein-coding genes, structural RNAs (5S, 16S, 23S), tRNAs and small non-coding RNAs were predicted and achieved by using the NCBI Prokaryotic Genome Annotation Pipeline (PGAP) server online [27]. The functional annotation of predicted ORFs was performed using RPS-BLAST [28] against the cluster of orthologous groups (COG) database [29] and Pfam database [30]. TMHMM program was used for gene prediction with transmembrane helices [31] and signalP program was used for prediction of genes with peptide signals [32].

## Genome properties

**Figure 3:**
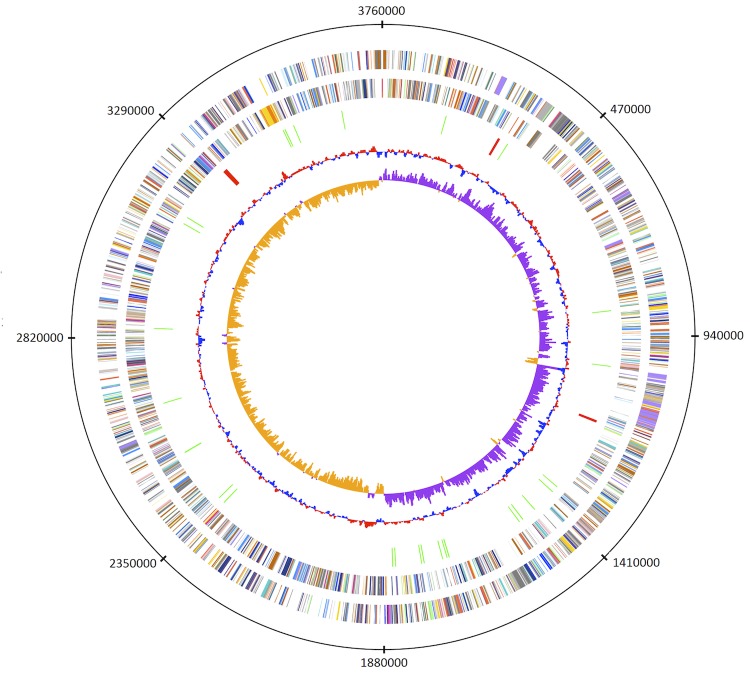
Graphical map of the chromosome. From outside to the center: Genes on forward strand (color by COG categories), genes on reverse strand (color by COG categories), RNA genes (tRNAs green, rRNAs red), GC content, GC skew.

The properties and the statistics of the genome are summarized in Table 4. The genome includes one circular chromosome of 3,764,053 bp (46.6% GC content). In total, 3,489 genes were predicted, 3,372 of which are protein-coding genes, and 61 RNAs; 56 pseudogenes were also identified. The majority of the protein-coding genes (67.07%) were assigned a putative function while the remaining ones were annotated as hypothetical proteins. The distribution of genes into COGs functional categories is presented in Table 5 and Figure 3.

**Table 4:** Genome statistics

**Attribute**	**Value**	**% of Total^a^**
Genome size (bp)	3,764,053	100.0
DNA coding region (bp)	3,315,444	88.08
DNA G+C content (bp)	1,753,947	46.60
Number of replicons	1	
Extrachromosomal elements	0	
Total genes	3,489	100.00
RNA genes	61	1.75
tRNA genes	48	1.38
rRNA operons	4	
ncRNA genes	1	0.03
Protein-coding genes	3,372	96.65
Pseudo genes	56	1.61
Genes with function prediction	2,340	67.07
Genes in paralog clusters	1,051	30.12
Genes assigned to COGs	2,249	64.46
Genes assigned Pfam domains	2,576	73.83
Genes with signal peptides	338	9.69
Genes with transmembrane helices	775	22.21

^a^The total is based on either the size of the genome in base pairs or on the total number of protein coding genes in the annotated genome.

**Table 5:** Number of genes associated with the 25 general COG functional categories

**Code**	**Value**	**%age**	**Description**
J	182	7.11	Translation, ribosomal structure and biogenesis
A	1	0.04	RNA processing and modification
K	161	6.29	Transcription
L	132	5.16	Replication, recombination and repair
B	1	0.04	Chromatin structure and dynamics
D	32	1.25	Cell cycle control, cell division, chromosome partitioning
Y	0	0.00	Nuclear structure
V	28	1.09	Defense mechanisms
T	152	5.94	Signal transduction mechanisms
M	150	5.86	Cell wall/membrane/envelope biogenesis
N	85	3.32	Cell motility
Z	1	0.04	Cytoskeleton
W	0	0.00	Extracellular structures
U	83	3.24	Intracellular trafficking, secretion, and vesicular transport
O	127	4.96	Posttranslational modification, protein turnover, chaperones
C	143	5.59	Energy production and conversion
G	76	2.97	Carbohydrate transport and metabolism
E	187	7.30	Amino acid transport and metabolism
F	67	2.62	Nucleotide transport and metabolism
H	115	4.49	Coenzyme transport and metabolism
I	106	4.14	Lipid transport and metabolism
P	138	5.39	Inorganic ion transport and metabolism
Q	57	2.23	Secondary metabolites biosynthesis, transport and catabolism
R	329	12.85	General function prediction only
S	207	8.09	Function unknown
-	1240	35.54	Not in COGs

## Comparisons with other *Thalassolituus* species genomes

Until now, only the genome sequence of the type strain *T. oleivorans* MIL-1^T^ was available within the genus of *Thalassolituus* [9]. Here, we compared the genome of strain R6-15 with strain MIL-1^T^ (Table 6). The genome of strain R6-15 is nearly 156 kb smaller in size than strain MIL-1^T^. The G+C content of strain R6-15 (46.6%) is similar with type strain MIL-1^T^ (46.6%). The gene content of strain R6-15 is smaller than strain MIL-1^T^ (3,489 vs 3,732).

**Table 6:** Comparison of genomes between *T. oleivorans* R6-15 and *T. oleivorans* MIL-1^T^

Genome Name	Genome size (bp)	Gene count	Protein coding	Protein with function	Without function	Plasmid number	rRNA operons
*T. oleivorans* R6-15	3,764,053	3,489	3,372	2,340	1,032	0	4
*T. oleivorans* MIL-1^T^	3,920,328	3,732	3,603	2,038	1,565	0	4

Strain R6-15 shares 2,995 orthologous genes with type strain MIL-1^T^. The average percentage of nucleotide sequence identity is 96.92% between strain R6-15 and MIL-1^T^. In addition, DNA-DNA hybridization (DDH) estimate value between strain R6-15 and MIL-1^T^ were calculated using the genome-to-genome distance calculator (GGDC2.0) [33,34]. The DDH estimate value between them was 84.5% ± 2.57, which were above the standard criteria (70%) [35]. Therefore, these results confirmed that strain R6-15 belonged to the species of *Thalassolituus oleivorans*.

## Conclusion

Strain R6-15 is the first strain with the complete genome sequence of the genus *Thalassolituus* isolated from the Arctic Ocean. These genomic data will provide insights into the mechanisms of how this bacterium can thrive on the crude oil in the polar marine environments.
